# Surgeon-Performed Saphenous Nerve Block at the Medial Femoral Condyle for Arthroscopic Partial Meniscectomy and Meniscus Repair: A Randomized Control Trial

**DOI:** 10.7759/cureus.26971

**Published:** 2022-07-18

**Authors:** Parker L Brush, Ruchir Nanavati, Gregory R Toci, Evan Conte, Joshua Hornstein

**Affiliations:** 1 Division of Sports Medicine, Rothman Orthopaedic Institute, Philadelphia, USA; 2 Orthopaedic Surgery, Rowan University School of Osteopathic Medicine, Stratford, USA

**Keywords:** opioid use, pain, local anesthesia, saphenous nerve block, knee arthroscopy

## Abstract

Introduction

With the rising number of arthroscopic partial meniscectomy and meniscus repair procedures performed in outpatient surgical centers, there has been significant interest in limiting postoperative pain and optimizing recovery. Postoperative pain is a common reason for admission at these surgical centers, and opioid-related mortality is becoming an increasing concern. A surgeon-performed saphenous nerve block (SNB) represents a promising adjunct treatment option for pain control. The purpose of this randomized controlled trial was to determine if an SNB would result in decreased postoperative pain and opioid usage compared to control following arthroscopic meniscus repair or partial meniscectomy.

Methods

We randomized patients between two groups: one receiving an SNB and the other without an SNB. The operating surgeon performed the SNB using a landmark-based approach at the medial femoral epicondyle/adductor hiatus with 5 mL of 1% lidocaine preoperatively and 5 mL of 0.5% ropivacaine postoperatively. Neither ultrasound nor nerve stimulator was utilized to confirm the success of the block. The visual analog scale was utilized to record pain preoperatively and in the immediate postoperative period, one day, and seven to 10 days postoperatively. The nursing staff in the post-anesthesia care unit monitored patient pain levels and provided pain medication accordingly.

Results

We enrolled 80 patients, with 40 patients in each group. There was no difference in age, sex, body mass index, or laterality between study groups. Overall, there were no differences between groups in preoperative or postoperative pain at any time. The average pain scores preoperatively were 2.78 in the experimental group and 3.05 in the control group (p=0.502). In the immediate postoperative period, pain scores were 1.57 for the experimental group and 2.66 for the control group (p=0.090). No statistically significant difference was detected in the number of patients requiring opioids postoperatively or in the morphine milligram equivalents (MME) provided to patients receiving opioids. Twelve patients in the experimental group received opioids in the immediate postoperative period while 18 patients in the control group received opioids (p=0.248). We observed no adverse events in patients from either group.

Conclusion

As a pure sensory nerve, the saphenous nerve is an excellent target for pain control without associated leg weakness. We utilized a low-resource SNB in knee arthroscopy for partial meniscectomy and meniscus repair as an adjunct therapy for postoperative pain control. This randomized controlled trial suggests that surgeon-performed blocks via a landmark-based approach are not effective in controlling pain or limiting opioid use in the immediate postoperative period. However, given our lack of confirmatory testing via ultrasound or nerve stimulation, a true nerve block may not have been achieved in all patients. We believe this adds to the external validity of this study, as these tools may not be readily available in all settings.

## Introduction

Arthroscopic knee surgery is routinely performed on an outpatient basis, and these cases contribute to a large portion of the volume at ambulatory surgery centers [1]. Pain control for these surgeries is managed in various ways, including nerve blocks, oral medications, and intra-articular medications [2]. In addition to improving patient satisfaction, improved pain control also hastens recovery and can lead to fewer days off of work [3]. In the immediate postoperative period, pain is a common reason for admission following surgery at an ambulatory surgery center [4]. Patients undergoing soft tissue-based arthroscopic knee surgeries, like meniscus repair, at ambulatory surgery centers require unplanned admission in approximately 5% of cases with postoperative pain as the most common cause [5].

In 2009, Volkow et al. found orthopedic surgeons to be responsible for 7.7% of all opioid prescriptions, however, in more recent years, Medicare trends suggest a decrease in opioid prescriptions by orthopedic surgeons [6-7]. As awareness of opioid-related mortality grows, surgeons are looking for alternatives to opioid prescriptions, including oral non-steroidal anti-inflammatory drugs (NSAIDs) [8-9]. A recent meta-analysis identified perioperative non-opioid pharmacotherapy, SNB, and cryotherapy as effective ways to reduce postoperative opioid use in the first 24 hours [10].

With regards to nerve blocks for pain control in knee arthroscopy, researchers have described techniques for blocks of the femoral, sciatic, obturator, and saphenous nerves, mainly through the assistance of ultrasound or a nerve stimulator [11-14]. In 2013, Hsu et al. conducted a randomized control trial regarding ultrasound-guided SNB in knee arthroscopy. They found decreased pain scores in their experimental group for the immediate postoperative period and did not observe quadriceps weakness and its inherent fall risk associated with femoral nerve blocks [14-15].

We identified the SNB as an effective alternative form of pain control for knee arthroscopy based on our literature review. However, compared to previous studies, we wanted to evaluate a low-resource landmark-based approach to the SNB without confirmatory ultrasound or nerve stimulator against a simple postoperative portal site injection of local anesthetic. We hypothesized the landmark-based approach to an SNB would result in decreased immediate postoperative pain scores as well as decreased opioid use in the immediate postoperative period when compared to patients receiving only local anesthetic at the portal sites.

## Materials and methods

After obtaining approval from the institutional review board at Thomas Jefferson University (approval number #20D.975), we conducted a randomized control trial with two groups: patients receiving an SNB in the experimental group and patients not receiving an SNB in the control group. We selected patients 18 years or older with the ability to consent to the research protocol undergoing meniscus repair or partial meniscectomy at one of our ambulatory surgical centers and excluded those who had prior open or arthroscopic surgery on the ipsilateral knee. Data collection occurred at ambulatory surgical centers owned by a single, private institution with operations performed by two fellowship-trained sports surgeons. We followed the recommendations found in the CONSORT 2010 statement in reporting this study [16].

After induction of general or sedation anesthesia and prior to portal placement, the primary surgeon performed an SNB with 5 mL of 1% lidocaine by a landmark-based approach at the medial femoral epicondyle/adductor hiatus for the experimental group. The control group did not receive an SNB. The surgeon also injected 5 mL of 1% lidocaine without epinephrine into each of the proposed portal sites for both groups. They performed the remainder of the procedure identically between the two groups, and after surgery, they injected 0.5% ropivacaine at the portal sites for both the control and experimental groups until a wheel was formed. The study group also received a final injection of 5 mL of 0.5% ropivacaine into the region of the saphenous nerve as described above.

Our primary outcomes were the immediate postoperative pain level by visual analog scale (VAS) and postoperative opioid use, and our secondary outcomes were postoperative Day 1 and Day 7 to 10 VAS. The primary surgeon obtained preoperative, immediate postoperative, one-day postoperative, and seven to 10-day postoperative pain levels by VAS. The nursing staff in the post-anesthesia care unit offered the patients pain medication and provided the medication per the patient's request. We converted postoperative opioid use into MME.

Our power analysis prior to the initiation of the study suggested that 160 subjects would be required to detect a meaningful difference in the VAS pain scale with a power of 80% and a significance level of p = 0.05. This analysis assumed a VAS pain score of 3.5 ± 2 in the SNB group based on previous studies [13-14]. We assigned each patient an opaque envelope during the consent process, and the envelope was provided to the surgeon at the time of operation to determine treatment group allocation. As a result, we were able to blind the patient to their treatment allocation, but the surgeon performing the block was not blinded to the allocation. We de-briefed the patients regarding their allocation after the study during the postoperative Day 7 to Day 10 follow-up.

All statistical analyses were done using R Studio (Version 3.6.3, Vienna, Austria). P-values for continuous data were calculated by t-tests or Mann-Whitney tests, and p-values for categorical data were calculated by chi-square or Fisher’s exact tests.

## Results

We recruited patients from January 2021 to April 2022 and recruitment was stopped early after the results of a preliminary statistical analysis with 80 patients. All 80 patients received their intended treatment with 40 in each group, and all patients were included in the final analysis.

The patients in the experimental group were not statistically different from the control group (Table 1) with regard to age (p=0.122), gender (p=1.00), BMI (p=0.118), laterality of procedure (p=0.642), history of diabetes (p=0.615), history of psychiatric disorders (p=0.737), history of chronic pain conditions (p=1.00), history of osteoarthritis (p=1.00), and history of rheumatoid arthritis (p=1.00). We searched for conditions such as fibromyalgia, complex regional pain syndrome, and chronic fatigue syndrome when identifying patients with chronic pain conditions and identified zero patients with such conditions. We did not identify any adverse reactions in the experimental or control groups.

**Table 1:** Demographics of the saphenous nerve block and control study groups

	Control (n=40)	Saphenous Nerve Block (n=40)	P-value
Age (years)	53.6 (9.42)	50.2 (12.1)	0.230
Sex (female)	9 (30.0%)	12 (40.0%)	0.417
Body mass index	29.6 (5.47)	27.9 (4.83)	0.118
Laterality (right)	13 (32.5%)	16 (40.0%)	0.642

On average, the patient-reported preoperative pain scores by VAS were 2.78 in the experimental group and 3.05 in the control group (p=0.502). In the immediate postoperative period, the patients in the experimental group rated their pain at 1.57 on average while the patients in the control group rated their pain as 2.66 on average (p=0.090). We identified no statistically significant difference in pain scores between the groups, including Day 1 (p= 0.331) and Days 7 to 10 (p=0.561) pain scores (Table 2).

**Table 2:** Pain scores and opioid use of the saphenous nerve block and control study groups

	Control (n=40)	Saphenous Nerve Block (n=40)	P-value
Opioid medications postoperatively	18 (45.0%)	12 (30.0%)	0.248
Morphine milligram equivalents	8.97 (3.98)	7.04 (4.00)	0.286
Visual Analog Score			
Preoperative	3.05 (2.34)	2.78 (2.50)	0.502
Immediate postoperative	2.66 (2.80)	1.57 (2.15)	0.090
1-day postoperative	3.2 (2.36)	2.67 (2.16)	0.331
7-day postoperative	1.75 (1.77)	1.96 (1.81)	0.561

Regarding immediate postoperative opioid use in the recovery room, 12 patients in the experimental group were given opioid pain medication and 18 patients in the control group were given opioid pain medication (Table 2). This difference was not found to be statistically significant (p=0.248). For patients who received opioids, we found no statistically significant difference in the MME provided between groups with 7.04 equivalents in the experimental group and 8.97 equivalents in the control group (p=0.286).

Our preliminary statistical analysis suggested that we would require an additional 168 patients (84 per group) to make a conclusion without committing type I or type II errors with regard to the immediate postoperative pain score. An additional 134 patients (67 per group) would be required for the same reasons to make a conclusion regarding the MME provided.

## Discussion

As a pure sensory nerve, the saphenous nerve represents an excellent target for pain control without associated leg weakness [17]. Prior researchers have published on the efficacy of an SNB for pain control after arthroscopy, but all of them utilized either ultrasound or nerve stimulation to confirm the placement of the block. Their results were mixed regarding the block's effect on postoperative pain scores and opioid consumption [13-14,18-19]. In 2020, Gazendam et al. performed a meta-analysis of these studies and included two additional studies [10]. One of these utilized a saphenous and posterior obturator nerve block and the other study was published in Spanish. Cumulatively, the SNBs performed in these studies were found to decrease oral MME and postoperative VAS pain scores at 24 hours when compared to controls without the blocks. These individual studies included a range from 40 to 73 patients [12-14,18-20].

Our study is different in that we have a larger study population of 80 patients, and our SNB was performed without confirmatory testing by ultrasound or nerve stimulation. Thus, representing a more feasible option in resource-limited settings and a less time-consuming process. It is our experience that the block performed in this study had a negligible effect on the operative time, with each block taking less than one minute to perform. Additionally, no training beyond that received in our fellowships was needed to perform this block. In 2005, Benzon et al. described a technique for an SNB at the medial femoral condyle by injecting an anesthetic in a fan-wise direction between the skin and the periosteum of the medial condyle [21]. Kent et al. prospectively compared the success rates of SNBs by the ultrasound-guided approach to the landmark-based approach described by Benzon et al. They found the landmark-based approach to be successful 30% of the time by pinprick evaluation, compared to 80% and 100% success rates for two different ultrasound-guided techniques [22]. We performed no tests to confirm the success of the SNBs, as ours were performed after general or sedation anesthesia, and although our landmark-based approach may have differed from the approach described in Benzon et al., we expected the success rate of our approach is less than that of an ultrasound-based approach. Given that our study found no statistical significance with regards to pain reduction, this potential decreased success of the approach may have played a key role when compared to other studies that found significant pain reduction [13-14,19].

The immediate postoperative pain scores by VAS in our study were low in comparison to these similar previous studies at 1.57 and 2.66 in the experimental and control groups, respectively. Akkaya et al. found postoperative pain scores of 3.15 and 5.25 in their SNB and control groups, respectively, and the postoperative pain scores in the study by Hsu et al. were 3.3 and 4.5 for the SNB and control groups, respectively [13-14]. However, the study by Hanson et al. described postoperative pain scores of 1.71 and 3.25 in their SNB and control groups, respectively. Unique to their study, an injection of bupivacaine was performed at the portal sites prior to the placement of surgical dressing for all patients [19]. In our study, lidocaine was injected prior to portal placement, and we used a ropivacaine injection after surgical closure of the portal sites. The studies by Akkaya et al. and Hsu et al. do not describe local portal site anesthetic injections [13-14]. As expected, we noticed a trend in these studies of decreased overall pain scores when the local was injected at the portal sites at the end of the surgery. Although these other studies were not designed to study the efficacy of the portal site injections of local anesthetic, our study was designed to evaluate the SNB in addition to the commonly performed local portal site injection, and we found no significant differences in pain scores [23-25]. This finding differs from the study by Hanson et al. where the SNB group was found to have lower pain scores in the immediate postoperative period [19]. However, our study includes a higher number of participants with 80 compared to 48, and our SNB was not performed under ultrasound guidance.

Apart from patient satisfaction with decreased pain, one of our primary goals was to utilize the SNB to reduce postoperative opioid use. Researchers in previous studies were inconsistent with regard to the efficacy of the SNB in reducing opioid consumption. Akkaya et al. utilized patient-controlled anesthesia with tramadol and identified decreased tramadol use in their nerve block cohort over the first 24 hours after surgery [13]. In our practice, admission for 24 hours and patient-controlled anesthesia are not routine following arthroscopic meniscectomy. Esplund et al. described a decrease in opioid consumption within the first two hours after surgery but not between two and 24 hours. However, they used the median and interquartile range to compare their groups, which can be misleading [18]. Hanson et al., whose methods were most like ours, utilized a protocol to treat the pain until scores were less than four. They provided their SNB group with an average of 40 micrograms of fentanyl and their control group with an average of 62 micrograms of fentanyl for 12 and 18.6 MME, respectively. Oral oxycodone was also included in their postoperative protocol [19]. On average, we provided fewer MME in the post-anesthesia care unit and hypothesize this is related to our methodology of opioids being provided by shared decision-making between the nursing staff and patients.

We made the decision to stop recruiting patients early because of the infrequency at which opioids were provided and the minimal differences between MME provided between our two groups. Our preliminary analysis suggested that more patients would be required than described by our original power analysis, and we believe this is related to the decreased accuracy of a landmark-based approach to SNB as well as the efficacy of the postoperative portal site injections in controlling pain.

Our limitations include the early cessation of recruitment, the lack of surgeon blinding to patient allocation, the decreased success rate of a landmark-based approach to the SNB, no strict protocol for postoperative pain management, outpatient surgery setting, the inherent variability of pain scores, and recruitment at a single-center by two surgeons. However, we intended to sacrifice the internal validity of the study with a less strict protocol to improve the generalizability of our findings.

## Conclusions

Our study is the largest to date evaluating the efficacy of an SNB for arthroscopic knee surgery. These data suggest that the addition of an SNB without image guidance or confirmatory testing to a simple postoperative portal site injection of local anesthetic offers no benefit to postoperative pain scores or opioid use. We did not include information regarding long-term follow-up of patients receiving an SNB, and thus we cannot assess for complications beyond one week or variations in long-term patient outcomes.
